# Approaches to protect and maintain health care services in armed conflict – meeting SDGs 3 and 16

**DOI:** 10.1186/s13031-019-0186-0

**Published:** 2019-01-29

**Authors:** Philippa Druce, Ekaterina Bogatyreva, Frederik Francois Siem, Scott Gates, Hanna Kaade, Johanne Sundby, Morten Rostrup, Catherine Andersen, Siri Camilla Aas Rustad, Andrew Tchie, Robert Mood, Håvard Mokleiv Nygård, Henrik Urdal, Andrea Sylvia Winkler

**Affiliations:** 1 Centre for Global Health, University of Oslo, Oslo, Norway; 2 Norwegian Red Cross, Oslo, Norway; 3 Peace Research Institute Oslo, Oslo, Norway; 4 World Health Organisation, Aleppo, Syria; 5 Médecins Sans Frontières, Oslo, Norway; 6 Department of Acute Medicine, Ullevål University Hospital, Oslo, Norway; 7 Norwegian Ministry of Foreign Affairs, Oslo, Norway; 8 Syria Relief UK, Manchester, UK

**Keywords:** Conflict, Sustainable development goal, Health, War, Syria, Health services

## Abstract

The escalation of conflict in the Middle East coincides with an emerging trend of attacks on healthcare. Protection of health personnel, health services and humanitarian workers is no longer respected. This compromises the achievement of the United Nations Sustainable Development Goals 3 – towards health for all, and 16 – towards justice and peace. The Centre for Global Health at the University of Oslo, the Peace Research Institute Oslo and the Norwegian Red Cross co-organised a meeting exploring how conflict impacts health systems and potential solutions to protect and maintain health care services.

## Background

**Fig. 1:**
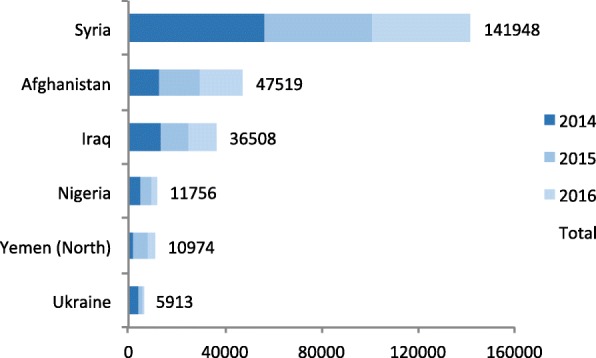
2014–2016 battle related deaths [17]

It is undisputed that “peace is essential … to ensure a healthy, productive global population” [1]. The United Nations Sustainable Development Goal (SDG) 16 aims to promote just, peaceful and inclusive societies, and SDG 3 aims to ensure healthy lives and promote wellbeing for all. Addressing the issue of attacks on health systems and personnel in situations of conflict is crucial to the achievement of both SDGs 3 and 16 [2]. War-related deaths have increased dramatically with the escalation of conflict in the Middle East [3]. In 2014, the Syrian Arab Republic recorded the highest number of battle-related deaths since 1989 (Fig. 1).

This increasing violence coincides with erosion of respect for the principles of International Humanitarian Law (IHL). Targeted attacks on health facilities have occurred in Syria, Yemen, Iraq, South Sudan and other conflict-affected countries in recent years. The United Nations (UN) Security Council’s Resolution 2286 was adopted in 2016 [4], strongly condemning attacks against medical facilities and personnel in conflict situations. If breaches are not met with strong sanctions, attacks on healthcare will continue with impunity. The Centre for Global Health (CGH) at the University of Oslo, the Peace Research Institute Oslo (PRIO) and the Norwegian Red Cross co-organised a meeting on November 29th 2017- “Approaches to protect and maintain healthcare services in armed conflict - meeting SDGs 3 and 16” exploring how conflict impacts health systems and exploring avenues to protect and maintain health care services in conflict settings. Challenges and potential solutions were discussed by medical doctors, humanitarian workers, scholars and diplomats.

### Challenges to protection, provision and maintenance of health services in armed conflict

#### Targeting of healthcare as an emerging tactic in conflict

Medical neutrality refers to a globally accepted principle derived from IHL, International Human Rights Law and Medical Ethics. It is based on principles of non-interference with medical services in times of armed conflict and civil unrest. It promotes the freedom for physicians and aid personnel to care for the sick and wounded, and to receive care regardless of political affiliations. IHL thereby condemns attacks on and misuse of medical facilities, transport and personnel.

**Fig. 2:**
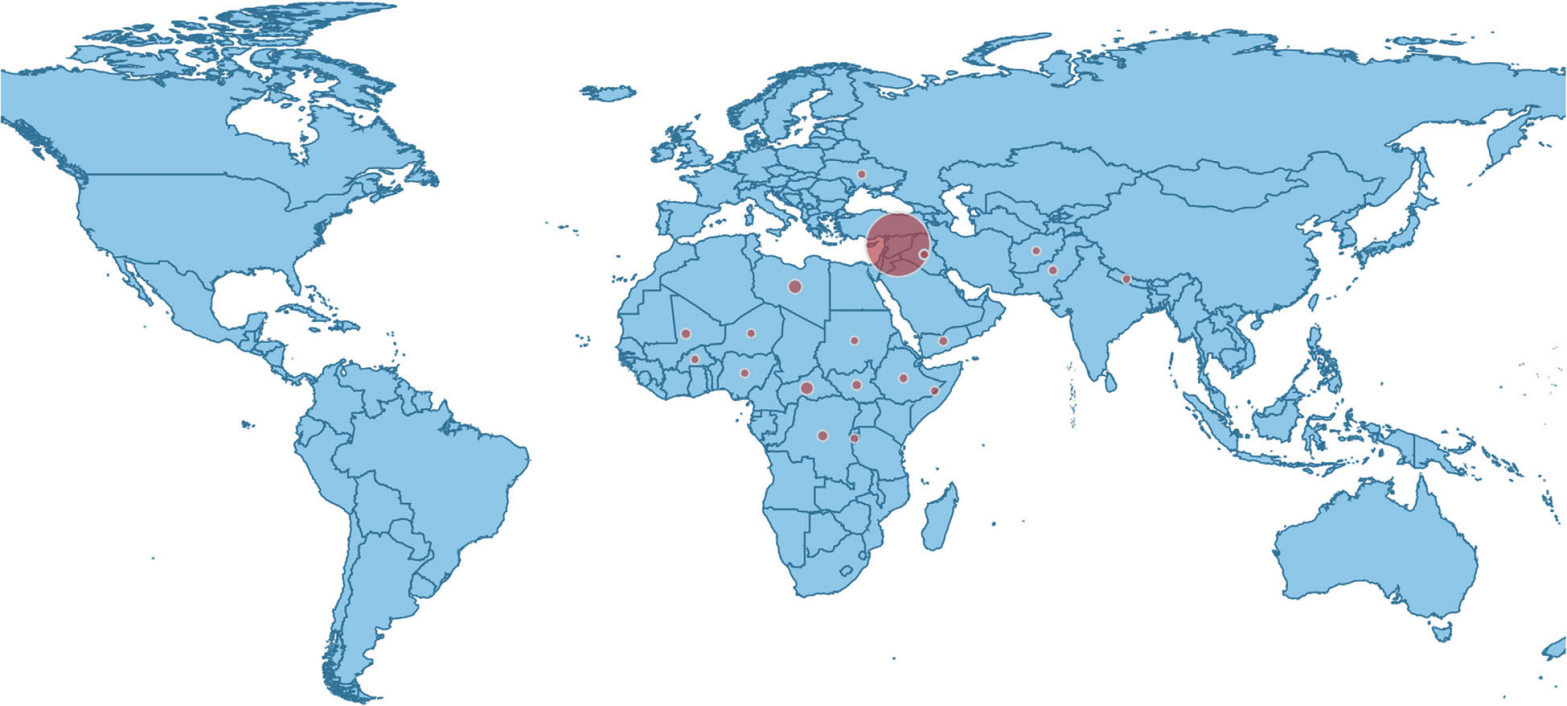
The number of attacks on health services in 2016 (highest occurring in Syria) [2]

Professor Scott Gates and Dr. Håvard Nygård presented data on the targeting of medical and humanitarian workers during conflict [2], also termed “irregular violence”. Since 2014, over 1500 medical workers have been attacked, many more have been threatened, injured, or suffered kidnapping and torture. The data show a relationship between irregular violence and battle casualties that occurred during and after 2014 (Figs. 1 and 2) - as conflict intensifies so too does the likelihood of attacks on health workers. Interestingly, data did not show any correlation between attacks on civilians and health or aid workers, implying that health workers are explicitly targeted.

With the changing climate of warfare and conflict, attitudes towards medical personnel in humanitarian missions have also shifted. Dr. Morten Rostrup described his experience with Médecins Sans Frontières (MSF) International. In the 1990s, Dr. Rostrup recalled the hospital was “the safest place to be in a conflict zone, because it was a respected place”. Dr. Rostrup observed a pivot in perception around 2001: while in Afghanistan a civilian asked if he was an American soldier, despite visibly wearing MSF clothing. Dr. Rostrup confirmed that armed groups now deliberately target medical staff and deny healthcare to specific populations.

#### Understanding the shift in respect for international humanitarian law

This erosion of respect for IHL in recent decades has complex explanations. One is that IHL is becoming more politicised, undermining its legitimacy. Some humanitarian actors may also be involved in peace negotiations or human rights advocacy, thereby undermining neutrality and impartiality [5]. Some state actions such as counterterrorism laws undermine IHL. States may also destabilise the humanitarian mission of non-governmental organisations (NGOs), exemplified by a 2001 speech by Colin Powell, former US Secretary of State, where NGOs were labelled as a conduit for the US military effort: “American NGOs are...an important part of our combat team” [6].

#### Consequences for health systems and local populations

Military strikes to health facilities result in profound acute- and long-term effects to health systems. Similarly, the strategic interruption of supply chains, electricity and water drastically impacts the capacity of health systems to deliver acute, preventive and routine care [7] [8]. Reduced capacity to address infectious disease is evident in Yemen where over one million suspected cholera cases have been recorded [9]. Frederik Siem from the Norwegian Red Cross highlighted that infant mortality rates usually increase by 13% during a typical five-year conflict [8] [10]. Indeed, the majority of deaths in conflict are caused by malnutrition and disease, dwarfing figures for deaths in battle [11].

Dr. Hanna Kaade, former World Health Organisation public health officer from Syria, reported that the majority of the health centres and major public hospitals in Syria were either destroyed and/or taken over by armed groups. He described the exodus of doctors early in the conflict due to threats, risk of kidnapping or death [12]. For patients, perceptions of danger can also translate into reduced care seeking. Professor Johanne Sundby recalled the UN Security Council resolution 1325 on Women, Peace and Security [13], emphasising that “violence against women not only increases and accelerates in war situations, but is used as a tactic against the civilian population.”

### Potential solutions to protect and maintain health systems

#### Strengthening international humanitarian law compliance

Catherine Andersen from the Norwegian Ministry of Foreign Affairs (MFA) underlined that parties to conflict have obligations to protect healthcare through IHL and to allow humanitarian systems to deliver during conflict. Frederik Siem highlighted the responsibility of states and armed groups to uphold IHL where they exercise territorial control, including providing health services or enabling access for alternative health service providers [14]. In raising awareness of the consequences of attacks on healthcare, Norway has built momentum to advocate for normative change. Partnerships with both international and professional organisations have been key to developing, promoting and embedding best practice for protection of healthcare in conflict settings, both prior to and during conflict.

Catherine Andersen encouraged vigilance to uphold IHL and active dialogue between states, funding bodies, health and humanitarian workers. Humanitarian actors need to be vocal if they are being instrumentalised by states in their work. Ongoing provision of health services in conflict settings varies according to the resilience of local systems and available humanitarian space. Dr. Rostrup recalled that IHL can only protect health and humanitarian workers insofar as they respect the principles of neutrality and impartiality. Field workers should be brave enough to hold humanitarian organisations accountable for upholding humanitarian principles.

#### Detecting and responding to attacks: Better data for global response

Panellists highlighted that humanitarian response requires quality conflict event data, including:

• Battle deaths & casualties, disaggregated by civilians or combatant status, health or humanitarian professionals

• Specific geographical and temporal data

• Onset of attack data (e.g. ‘double-taps’; hitting two targets in quick succession for maximum casualties)

• Health indicators e.g. maternal and infant mortality, disease incidence

• Strategies for protection of civilians and infrastructure

Academics have a role to improve data quality and analysis alongside humanitarian and state actors.

#### Investing in local systems to restore peace

Failure to protect civilians has long term consequences. Fear of violence results in exodus of doctors, teachers and civilians and affects the willingness of refugees to return. Breaches in IHL erode trust in a functioning society, thereby compromising the reconstruction process. The maintenance and reconstruction of health systems also serves a longer term purpose: to promote return and restore optimism for those remaining, affirmed Dr. Kaade. Development actors are now called on to engage in early intervention in conflict zones for peace and development [15, 16].

## Conclusion

Participants reinforced the urgency of addressing attacks on health systems and personnel, calling for strengthened compliance with IHL by states and armed groups alike. Strong mechanisms to investigate and sanction IHL breaches are necessary, as well as dialogue and cooperation between states, academic and humanitarian sectors. Better data is necessary in order to detect and analyse attacks, for appropriate response. Restoration of peace requires local health providers, functioning systems and services to address violence and its victims. Maintaining health systems in conflict settings requires investment in local systems to encourage populations to remain, return and rebuild. Protecting and rebuilding the health care system must be a priority during and after conflict in order to achieve both SDG 3 and 16. This meeting calls for state, humanitarian and academics sectors to collaborate closely in contributing to achieving SDGs 3 and 16.

## Abbreviations

CGH: Centre for Global Health, University of Oslo
IHL: International Humanitarian Law
MFA: Norwegian Ministry of Foreign Affairs
MISP: Minimum Initial Service Package for Reproductive Health
MSF: Médecins Sans Frontières (Doctors Without Borders)
NGO: Non-governmental organisation
PRIO: Peace Research Institute Oslo
SDG: Sustainable Development Goal
UN: United Nations
USA: United States of America

## References

[1] Wesley H, Tittle V, Seita A (2016). No health without peace: why SDG 16 is essential for health. Lancet.

[2] Gates S, Mokleiv Nygård H, Bahgat K. Patterns of attacks on medical personnel and facilities: SDG 3 meets SDG 16. 2017 [cited 2018 Jan 9]. Available from: https://www.prio.org/Publications/Publication/?x=10785.

[3] Human Development Report (2016). Human development for everyone. United Nations development Programme.

[4] Security Council Adopts Resolution 2286 strongly Condemning Attacks against Medical Facilities [press release]. Personnel in Conflict Situations 2016.

[5] Leebaw B (2007). The politics of impartial activism: humanitarianism and human rights. Persp on Pol.

[6] Powell CL. September 11, 2001: Attack on America remarks to the national foreign policy conference for leaders of non-governmental organizations 2001. Available from: http://avalon.law.yale.edu/sept11/powell_brief31.asp.

[7] ICRC (2016). Protracted conflict and humanitarian action: some recent ICRC experiences.

[8] Siem FF (2017). Leaving them behind healthcare services in situations of armed conflict. Tidsskrift for Den norske legeforening.

[9] Suspected cholera cases in Yemen surpass one million, reports UN health agency [press release]. UN News 2017.

[10] Breaking the conflict trap. World Bank; 2003.

[11] Human Security Report 2005: war and peace in the 21st century. Human Security Centre; 2005.

[12] Baker A. Syria’s health crisis spirals as doctors flee. Time. 2014:4.

[13] Security Council resolution 1325, (2000).

[14] Siem FF. Armed groups and Access to Health in Conflict Areas. Oslo, Norway: Norwegian Red Cross; 2015.

[15] Norway’s Humanitarian Strategy: an effective and integrated approach. Oslo: Norwegian Ministry of Foreign Affairs; 2018.

[16] The Humanitarian-Development-Peace Initiative: World Bank Group; 2017. Available from: http://www.worldbank.org/en/topic/fragilityconflictviolence/brief/the-humanitarian-development-peace-initiative.

[17] UCDP. Uppsala Conflict Data Program: Department of Peace and Conflict Research. 2018. Available from: http://ucdp.uu.se.

